# Are mesenchymal stromal cells immune cells?

**DOI:** 10.1186/s13075-015-0596-3

**Published:** 2015-03-31

**Authors:** Martin J Hoogduijn

**Affiliations:** Nephrology and Transplantation, Department of Internal Medicine, Erasmus MC, 3000 CA Rotterdam, the Netherlands

## Abstract

Mesenchymal stromal cells (MSCs) are considered to be promising agents for the treatment of immunological disease. Although originally identified as precursor cells for mesenchymal lineages, *in vitro* studies have demonstrated that MSCs possess diverse immune regulatory capacities. Pre-clinical models have shown beneficial effects of MSCs in multiple immunological diseases and a number of phase 1/2 clinical trials carried out so far have reported signs of immune modulation after MSC infusion. These data indicate that MSCs play a central role in the immune response. This raises the academic question whether MSCs are immune cells or whether they are tissue precursor cells with immunoregulatory capacity. Correct understanding of the immunological properties and origin of MSCs will aid in the appropriate and safe use of the cells for clinical therapy. In this review the whole spectrum of immunological properties of MSCs is discussed with the aim of determining the position of MSCs in the immune system.

## Introduction

Mesenchymal stromal cells (MSCs) were originally identified as precursors for cells of the osteogenic lineage [1]. They were later discovered to be able to differentiate also into the chondrogenic, adipogenic and myogenic lineages [2]. Within the scientific community there is some controversy about the naming and precise definition of MSCs. The term 'mesenchymal stromal cell' is used in parallel with 'mesenchymal stem cell' and 'multipotent mesenchymal stromal cell'. MSCs are in fact a heterogeneous population of cells that express CD73, CD90 and CD105 and lack the haematopoietic lineage markers CD45, CD34, CD11c, CD14, CD19, CD79A and HLA-DR [3]. This immunophenotype, however, covers various subsets of MSCs with different phenotypes and different functions [4,5]. Cell isolation procedures can, therefore, affect the cellular composition of MSC cultures. Culture conditions can have a further impact on the phenotype and function of MSCs [6]. This may affect study outcomes. Therefore, some care should be taken in comparing the results of studies using different MSC isolation and culture procedures.

In the bone marrow, MSCs have a supportive function for the haematopoietic system and provide a niche for haematopoietic progenitor cells to mature. The presence of MSCs is not limited, however, to the bone marrow and in other tissues, such as adipose tissue, muscle and multiple organs, they provide support for tissue cells by producing growth factors and matrix proteins. In addition to their differentiation and tissue supportive functions, MSCs have a well-established immune modulatory function. Several *in vitro* studies have demonstrated that MSCs are able to effectively inhibit T lymphocyte [7,8] and natural killer (NK) lymphocyte [9] proliferation, impair antibody production by B cells [10], and inhibit the maturation and function of dendritic cells [11]. Studies in animal models have shown that MSCs can reduce disease progression and/or severity of various immune diseases such as collagen-induced arthritis [12], experimental autoimmune encephalomyelitis [13], experimental colitis and sepsis [14]. It is believed that MSCs mediate their beneficial effects by modulating the immune system, although the exact mechanisms of immunomodulation by MSCs *in vivo* are not clear. Even though there is abundant evidence that MSCs modulate immune responses by interacting with cells of the immune system, the question is whether MSCs themselves should be perceived as true immune cells. Do MSCs exercise immune functions like immune cells do and what is their response to pathogens? In this review, the various immunological roles of MSCs are discussed, culminating in a conclusion on the position of MSCs in the immune system.

## Immunological properties of mesenchymal stromal cells

### Interaction with immune cells

**Figure 1:**
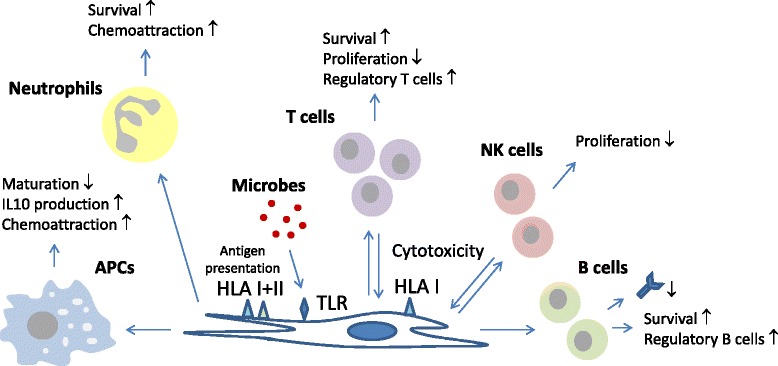
**Overview of the interactions between mesenchymal stromal cells (MSCs) and immune cells.** MSCs secrete cytokines (including transforming growth factor β, hepatocyte growth factor, IL-6, IL-7), chemokines (CCL2, IL-8) and prostaglandins (prostaglandin-E2), and express proliferation inhibitory cell surface molecules (programmed death ligand 1, Fas ligand) and metabolic enzymes (indoleamine 2,3-dioxygenase, CD73) that target immune cells in various ways. APC, antigen-presenting cell; NK, natural killer; TLR, Toll-like receptor.

MSCs interact with cells of the immune system via a plethora of mechanisms. They secrete anti-inflammatory factors such as transforming growth factor β (TGF-β), hepatocyte growth factor (HGF) and prostaglandin-E2 (PGE-2) [7,8], and they express cell surface molecules with immunosuppressive properties such as programmed death ligand 1 (PD-L1) and Fas ligand [15,16], via which they directly target immune cells and inhibit their activation and function. MSCs furthermore attract immune cells by secreting a broad mixture of chemokines. In particular, the neutrophil chemo-attractant interleukin (IL)-8 and the monocyte-attractant CCL2 are secreted in high amounts by MSCs [17]. Chemokine secretion by MSCs may act in a dual way to modulate the immune response. Reactive immune cells will be attracted and exert their immunological function, but at the same time they may be targeted by MSCs and inhibited in their function. There is evidence that MSCs bind activated immune cells [18], potentially to keep them at a close distance to enhance the effect of their immunosuppressive actions. The immunoregulatory effects of MSCs are not only directed directly against efxfector immune cells. MSCs do not themselves produce the anti-inflammatory cytokine IL-10, but they induce other cell types to do this [19]. Via the secretion of TGF-β and other factors MSCs also promote the induction of regulatory T cells [20], regulatory macrophages [21] and regulatory B cells [22], and in this way pass on their immunosuppressive effects to other cell types that exert different mechanisms of immune suppression. A schematic overview of the interactions between MSCs and immune cells is depicted in Figure 1.

### Metabolic control of the immune system

In addition to the well described mechanisms of immune modulation via cytokines, chemokines and inhibitory co-stimulation molecules, MSCs are actively involved in the metabolic control of the immune system. MSCs can be induced to express the enzyme indoleamine 2,3-dioxygenase (IDO), which has a potent capacity to inhibit lymphocyte proliferation by metabolising L-tryptophan to L-kynurenine [23]. Reduced levels of L-tryptophan suppress lymphocyte proliferation and, simultaneously, high levels of L-kynurenine impose a block on proliferation as well. Furthermore, MSCs strongly and constitutively express CD73 [3], which acts in concordance with CD39 on regulatory T cells to metabolise ATP to adenosine, thereby taking away the inflammation-promoting effect of ATP [24]. Metabolic control of the immune response also occurs through other mechanisms, such as L-arginine metabolism, which is employed by myeloid cells to inhibit lymphocyte activities [25]. It is unknown whether MSCs make use of this pathway to regulate immune cells.

### Adaptation to inflammatory conditions

MSCs can be considered as genuine regulators of the immune system because they adapt their immunoregulatory properties to the local immunological milieu. MSCs sense inflammation and in response to pro-inflammatory cytokines, in particular interferon-γ and tumour necrosis factor (TNF)-α, they change their immunological role. In the absence of inflammation, MSCs promote the survival of T lymphocytes [26] and can even stimulate their proliferation, partly via IL-6-dependent mechanisms [27]. MSCs have recently been identified as producers of IL-7 [28], which stimulates the differentiation of haematopoietic cells into lymphoid cells but also promotes the proliferation of lymphocytes. Once placed under immune activating conditions, MSCs upregulate the expression of IDO, HGF, PD-L1, TNF-stimulated gene 6 protein and PGE2-producing cyclooxygenase 2 [29] as part of immunological negative feedback loops [30]. MSCs adapted to inflammatory conditions will inhibit immune cell activation and proliferation [31] and increase their regulatory T cell-inducing capacity [32]. Even though the expression of pro-inflammatory factors such as IL-6, IL-7 and several chemokines by MSCs is also increased under inflammatory conditions, the immunosuppressive effects of MSCs prevail under these conditions. MSCs thus play a central role in maintaining immune homeostasis by interacting with immune cells via cytokines, chemokines, cell surface molecules and metabolic pathways. The question is whether this is sufficient to qualify MSCs as immune cells. Strictly speaking, an immune cell protects organisms against pathogens, cleans up cell debris, and removes diseased cells. The MSC functions described above steer the activities of the immune system but do not by themselves represent core immune functions. Do MSCs possess these properties?

## Role of mesenchymal stromal cells in immune defence

### Immune cell effector functions

Immune cells fight diseased cells and pathogens by cytotoxic activity, antibody production, and phagocytosis. MSCs do not express granzymes or perforins and do not produce antibodies and are not, therefore, capable of cytotoxic activity or participation in the humoral defence. There is some evidence, however, that MSCs possess phagocytic properties. It was shown that MSCs can phagocytise apoptotic cells, which as a result enhances their osteogenic differentiation capacity [33]. MSCs that phagocytised apoptotic cells increased chemokine expression and induced Th17 cells, suggesting that phagocytosis leads to an immune-activating response in MSCs. More data on the phagocytic activity of MSCs are not available at present, which leaves their role as a phagocytic cell unclear. Overall, there is no overwhelming evidence that MSCs act as effector cells in the immune system.

### Antigen presentation

Whereas MSCs may not have immune cell effector functions, they can play a role in the initiation of immune responses. MSCs have the capacity, like all nucleated cells, to present antigen via major histocompatibility complex (MHC) class I molecules, which are constitutively expressed on MSCs. Antigens presented via MHC class I are endogenous antigens and their presentation serves the recognition of pathogen-infected or transformed cells by CD8^+^ cytotoxic cells. Under inflammatory conditions, MSCs also express MHC class II and thereby gain the capacity to present exogenous antigens to T cells [34,35], a property shared by professional antigen-presenting cells such as dendritic cells and macrophages. Interferon-γ-stimulated MSCs furthermore possess MHC class II-mediated antigen processing capacity [36]. Under inflammatory conditions MSCs can thus present antigens from their environment and induce adaptive immune responses by activation of CD4^+^ T cells. In addition, MSCs can cross-present antigens via their MHC class I molecules and process antigen via proteasome- and transporter molecule-dependent mechanisms [37]. Via this route MSCs can initiate CD8^+^ T-cell responses to exogenous antigens. Although the ability to process and present antigens would appear to be a typical immune cell function, is it not unique to immune cells. Endothelial cells and fibroblasts, like MSCs, upregulate MHC class II under inflammatory conditions, and under these conditions they are potent stimulators of CD4^+^ T-cell responses [38]. Endothelial cells furthermore have the capacity to cross-present antigens via MHC class I [39]. Antigen presentation under inflammatory conditions is thus a capacity shared by different types of tissue cells.

### Response to pathogens

MSCs do not possess receptors that recognise specific antigens, such as cells of the adaptive immune system do via T- and B-cell receptors. Recognition of antigen by innate immune cells is mediated via a broad range of pattern recognition receptors. MSCs express certain pattern recognition receptors, including NOD-like receptors [40] and Toll-like receptors (TLRs) [41]. Inflammatory conditions affect the expression of TLRs on MSCs [42] and TLR activation may lead to an inhibition of the immunosuppressive effects of MSCs, allowing T-cell responses to build up [43], although there is also evidence that the immunosuppressive effects of MSCs are increased by TLR activation [44]. Via their pattern recognition receptors MSCs recognise microbes and upon MSC-microbe association they increase the expression of immunomodulatory genes such as IL-6, IL-8 and cyclooxygenase-2 [45]. As a result, the capacity of MSCs to inhibit T-cell proliferation is enhanced, which could serve as a negative feedback loop to protect against collateral damage of strong immune responses against microbes. MSCs furthermore exert direct anti-microbial effects, as demonstrated in *Escherichia coli*-injured human lungs. MSCs were shown to phagocytise bacteria and secrete keratinocyte growth factor, which induces monocytes to support the anti-microbial effect of MSCs [46]. MSCs do thus participate in the defence against microbial threats.

### Migration to sites of inflammation

Immune cells migrate to sites of infection/inflammation in response to chemokine attraction. Upon activation they upregulate adhesion molecules and rolling and invasion machinery and stick to the endothelium and migrate in between endothelial cells and tissue cells to the source of the chemokine production. MSCs also express chemokine receptors and migrate *in vitro* in response to chemotactic stimulation. This property is enhanced under inflammatory conditions [47]. There is controversy about the migratory ability of *in vivo* administered MSCs, but for a comparison of the migratory properties of MSCs with immune cells the migration of endogenous MSCs should be discussed. Whereas MSCs have been detected in the circulation in animal models, there is little evidence for the presence of MSCs in the human circulation [48], except for under conditions where the MSC niche is disrupted, such as in trauma patients [49,50]. A study has demonstrated that human MSCs can egress from adipose tissue to migrate to lymph nodes [51], suggesting that MSCs may avoid the blood stream as a means of transportation and instead use the lymphatic system. Via the lymphatic system MSCs would not, however, reach sites of tissue inflammation. In contrast to neutrophils, macrophages and lymphocytes, the need for MSC recruitment from distant sites to inflamed tissue may not be essential as MSCs are already present in all tissues. MSCs may be recruited locally to increase their presence at inflamed sites.

MSCs thus certainly play a role in the immune defence, but their tasks are not as specialised as those of other types of immune cells. MSCs rather support various aspects of the immune response.

## The origin of mesenchymal stromal cells

### Mesenchymal origin

During embryonic development MSCs are derived from the mesodermal germ layer; the mesoderm forms the connective tissue and the haematopoietic system. Whereas all traditional immune cells are of haematopoietic origin, the developmental origin of MSCs is not entirely clear and a matter of debate. The hypothesis that MSCs are of bone marrow stromal origin and migrate from there to peripheral tissues to occupy their place as regenerative and immunomodulatory cells during adulthood is outdated by data showing that MSCs of recipient origin are not found in transplanted organs even many years after transplantation [52] and the absence of MSCs in the circulation of healthy individuals and patients with severe organ injury [50]. The discovery in multiple organs of cells around blood vessels that lack haematopoietic, endothelial, and myogenic cell markers but possess multi-lineage differentiation capacity and express MSC markers has suggested that MSCs are of perivascular origin [53]. More recently, it was demonstrated that tooth MSCs are derived from peripheral nerve-associated glial cells [54]. These results suggest that MSCs are distributed throughout the body during development and reside in their specific niche during adulthood from where they act locally to mediate regenerative and immunomodulatory processes.

### Haematopoietic origin?

Although the non-haematological origin of MSCs is generally accepted, data suggest that the distinction between haematopoietic and non-haematopoietic cells may not be as sharp as commonly believed. A suggestion for this may arise from the fact that adipose tissue-derived MSCs express the haematopoietic stem cell marker CD34. Furthermore, elegant experiments carried out a decade ago demonstrated that the transplantation of single green fluorescent protein (GFP)-expressing haematopoietic stem cells in mice led to the generation of GFP-positive microglial and perivascular cells [55], cells that are members of the MSC family. This would indicate that primitive haematopoietic progenitor cells have the capacity to differentiate into mesenchymal lineages. In further support of the relationship of MSCs with the haematopoietic lineage are the similarities that exist between fibroblasts and macrophages, as reviewed by Ogawa and colleagues [56]. Fibroblasts are derived from MSCs and like their precursor cells they can adapt immunoregulatory properties and become activated to secrete growth factors in case of tissue injury. Macrophages in their turn can adapt similar regenerative properties and can home to injured tissue where they stimulate repair processes [57]. In contrast to the idea that MSCs are of haematopoietic origin, it is well known that MSCs can be locally formed in tissue in a process called epithelial to mesenchymal transition in which epithelial cells give rise to MSCs in response to injury. Despite the so far contrasting and insufficient data on the classification of MSCs, the view that MSCs are of non-haematopoietic origin is currently the most widely accepted. In this view, MSCs are thus of a different lineage than classical immune cells.

## Immunomodulatory therapy with mesenchymal stromal cells

### Infusion of mesenchymal stromal cells

The use of MSCs for immunomodulatory therapy for a variety of immunological disorders is intensively studied. Phase 1/2 clinical trials have been performed in graft versus host disease [58], organ transplantation [59,60], and multiple types of autoimmune diseases, including inflammatory bowel disease [61,62], systemic lupus erythematosus [63,64] and multiple sclerosis [65]. Some of the studies showed amelioration of disease severity, albeit the studies were non-controlled. The use of MSCs for rheumatoid arthritis has been examined in disease models by multiple research groups [66] and a clinical study in 172 rheumatoid arthritis patients demonstrated that intravenous infusion of allogeneic MSCs was feasible and safe and induced a significant disease remission [67]. A placebo-controlled study in chronic obstructive pulmonary disease demonstrated that MSC therapy lowered C-reactive protein levels, but did not affect disease indicators [68]. Although the efficacy of MSC immunotherapy remains to be demonstrated in larger placebo-controlled trials, in several of the studies there were indications that the infusion of MSCs leads to immunomodulatory effects. Do these effects prove that MSCs are immune cells? If one looks in detail at the immunological effects that are induced by the infusion of MSCs, it can be observed that MSCs induce small inflammatory responses shortly after infusion [69], whereas it is likely that the immunosuppressive effects of MSCs take longer to occur. MSCs that are administered via the intravenous route are, however, short-lived and the large majority of them disappear after 24 hours [70]. It is thus likely that the immunosuppressive effects of MSC treatment are mediated by other cell types and there is indeed accumulating evidence that MSCs induce regulatory T cells [71,72]. Thus, MSCs themselves may not be active as immunoregulators after administration, but the regulatory immune cells that they induce may mediate these effects. In this sense, MSCs do not fit the definition of an immune cell, but should rather been seen as coordinators of the immune system.

Basically all clinical studies of the immunomodulatory effect of MSCs have been performed after intravenous infusion of MSCs. Intravenous infusion is the easiest and therefore most commonly used route of administration of MSCs, but it is possible that MSCs that are administered via other routes act via different modes of action. When MSCs are administered intramuscularly or are delivered via the arterial route to tissues of interest, they localise close to or even within sites of inflammation and they may interact with immune cells in a more direct way and may survive for a longer time. Whether MSCs administered into inflammatory sites act more like true immunoregulatory cells needs to be studied in more detail.

### Immunogenicity of mesenchymal stromal cells

In contrast to immune cells, MSCs express low levels of HLA class I and co-stimulatory molecules CD80 and CD86 and are therefore low immunogenic. Like other cells, MSCs do, however, induce allogeneic immune cell responses, as demonstrated by the lysis of MSCs by HLA class I mismatched memory CD8^+^ T cells [73]. In this sense MSCs do not behave differently from immune cells. Different from immune cells, culture-expanded MSCs are also susceptible to lysis by autologous IL-2-activated NK cells [9]. Lysis by NK cells depends on the relatively low expression of HLA class I molecules on MSCs and the expression of activating NK cell receptor ligands. Lysis by autologous NK cells indicates that *in vitro* expansion induces the immunogenicity of MSCs and, despite all their immune regulatory properties, makes them targets of the immune defence themselves.

## Conclusion

MSCs play a core role in maintaining immune homeostasis in their niche in most, if not all, tissues by interacting with antigen-presenting cells, phagocytic cells, cytotoxic cells, B cells and helper T cells via soluble and cell membrane-mediated mechanisms. This property is maintained by cells of more differentiated mesenchymal lineages, such as fibroblasts. Under inflammatory conditions MSCs gain additional immunological functions, such as antigen presentation. After culture expansion and administration in humans or animals, MSCs acquire a different function and trigger immunomodulatory responses by their short presence. Their developmental origin and the limited migratory properties of MSCs, which are associated with their role as precursor cells for mesenchymal cells within tissues, show that MSCs are not true immune cells. They are nevertheless omissible for controlled functioning of the immune system and there are promising prospects for the development of MSC-based immune therapy in the near future.

## Note

This article is part of a thematic series on *Biology and clinical applications of stem cells for autoimmune and musculoskeletal disorders*, edited by Christian Jorgensen and Anthony Hollander. Other articles in this series can be found at http://www.biomedcentral.com/series/MSC

## Abbreviations

GFP: green fluorescent protein
HGF: hepatocyte growth factor
IDO: indoleamine 2,3-dioxygenase
IL: interleukin
MHC: major histocompatibility complex
MSC: mesenchymal stromal cell
NK: natural killer
PD-L1: programmed death ligand 1
PGE-2: prostaglandin-E2
TGF-β: transforming growth factor β
TLR: Toll-like receptor
TNF: tumour necrosis factor
